# Enhanced Cell Proliferation, Migration, and Fibroblast Differentiation with Electrospun PCL–Zinc Scaffolds Coated with Fibroblast-Derived ECM

**DOI:** 10.1021/acsomega.4c07504

**Published:** 2025-01-28

**Authors:** Alexis Moody, Narayan Bhattarai

**Affiliations:** † Department of Applied Science and Technology, North Carolina A&T State University, Greensboro, North Carolina 27411, United States; ‡ Department of Chemical, Biological, and Bioengineering, North Carolina A&T State University, Greensboro, North Carolina 27411, United States

## Abstract

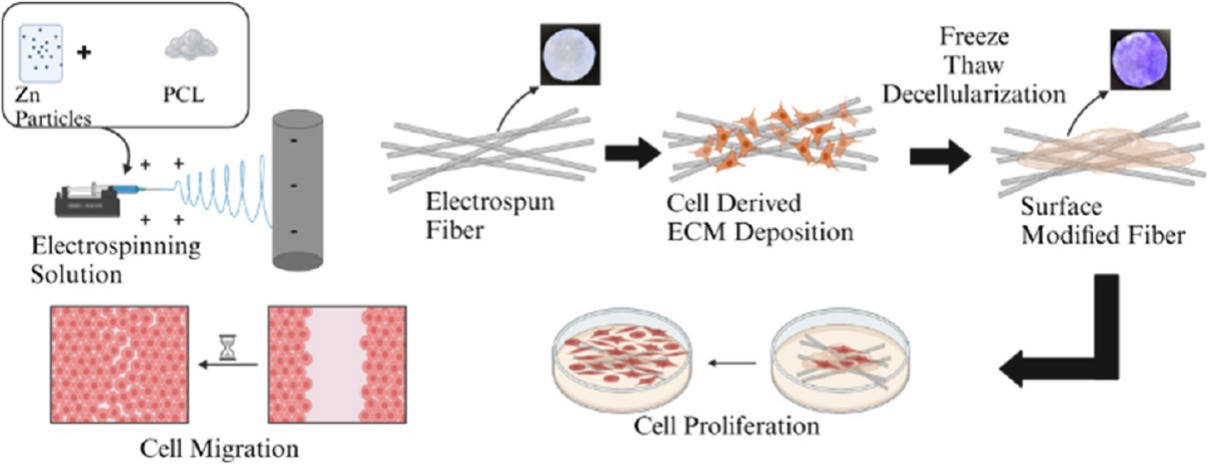

Despite tremendous improvement in the development of tissue-regenerating materials, a promising solution that provides an optimal environment remains to be accomplished. Here, we report a composite nanofiber biomaterial scaffold as a promising solution that closely mimics the extracellular matrix (ECM) to improve cell viability, proliferation, and migration. Initially, nanofiber composites of polycaprolactone (PCL) and zinc (Zn) metal were fabricated by using electrospinning. The resulting PCL–Zn (PZ) nanofibers effectively guided the growth of NIH3T3 fibroblasts for 7 days, forming a fibroblast cell sheet. The PZ fibers were decellularized to remove autologous and allogenic cellular antigens while leaving an intact ECM with structural and functional components. The resulting nanofiber PCL–Zn–ECM (PZE) showcased a natural ECM bonded to the surface, providing a bioactive element to the interconnected fibers. The reseeding of NIH3T3 fibroblasts demonstrated the scaffold’s excellent capacity to direct and support cell proliferation. Furthermore, in vitro cytotoxicity analysis and morphological staining confer the scaffold’s biocompatibility. The PZE scaffold presents a promising development in which these scaffolds can be further used for various regenerative medicine applications including wound healing.

## Introduction

Biomaterial scaffolds play an important role in tissue engineering by providing support for seeded cells until they are organized into functional tissue.^1^ These scaffolds are designed to guide regeneration by providing a framework for cells to adhere, proliferate, and differentiate^2^ Recently, there has been a growing interest in tailoring scaffold properties to better mimic the natural extracellular matrix and enhance overall tissue regeneration outcomes for applications like chronic wound healing.^3−15^ However, several challenges remain to be addressed including optimized interactions between biomaterials and cells, continual refinement of biomaterial properties, risks of immunogenicity, and issues related to patient compliance.^16−18^ Consequently, it is critical to pick appropriate scaffold materials based on an extensive understanding of biological mechanics. In tissue engineering practice and wound healing therapy, biocompatible polymeric scaffolds are used to stimulate cell growth and proliferation.^8,19,20^ Polycaprolactone (PCL) is a polymer that is predominately used for biomedical applications because it has a high degree of solubility in various solvents, degradation ability, and biocompatibility.^21,22^ Yet, the major disadvantages of PCL include hydrophobicity and poor bioactivity, which negatively affects cell adhesion, restricts cell attachment, and decreases cellular proliferation.^23,24^ Several methods have been introduced to overcome the shortcomings to increase cell viability including blending PCL with more hydrophilic materials and various surface coating techniques.^24−26^

Studies show that some metal elements, which are necessary components of the human body, influence the regulation of cytokines and growth factors in the processes of cell proliferation and migration.^27,28^ Nondegradable metals like silver (Ag) have been used for their antimicrobial properties and ability to reduce inflammation for centuries.^29,30^ Meanwhile, degradable metals, such as magnesium (Mg) and zinc (Zn), can also have a positive effect on cellular functions. Mg is essential for cell proliferation, cell cycle, and protein synthesis.^31^ Zn, an essential trace element, plays roles in cell membrane repair, cell proliferation, and immune system function.^32^ While daily oral supplementation of 18–20 mg of Zn has been shown to significantly reduce chronic ulcer size after 12 weeks of supplementation and topical treatment using aqueous solutions containing 0.2 mg/100 mL per 10 cm^2^ wound significantly improved healing in the wounds of diabetic patients, more studies are needed to fully understand its role in enhancing cell proliferation and migration.^33−35^

Due to their positive effects on enhancing cell proliferation and migration, metals such as Mg and Zn have been considered for incorporation into scaffolds for clinical applications.^36^ Metal-based composite biomaterial scaffolds have been shown to support biocompatibility and cell proliferation.^37^ Composite metal/polymer biomaterials also regulate cell behavior and function as controlled release systems for continuous aid for cell growth.^38,39^ For example, Mg/PCL scaffolds exhibited cytocompatibility for NIH3T3 fibroblasts and PC-12 pheochromocytoma cells while also exhibiting anti-inflammatory properties in mice.^39^ Yang et al. designed a GelMa hydrogel loaded with Zn and Mg particles to support cell proliferation and tissue regeneration. The GelMA/Mg/Zn release increased bioactivity, induced fibroblast differentiation by activating the STAT3 signaling pathway, and accelerated collagen deposition in rats.^40^

Similar to metal-based composites, materials derived from naturally occurring extracellular matrix (ECM) proteins also aim to support cellular activities. The ECM provides functionality and structural integrity while controlling fundamental cell behaviors like proliferation, migration, adhesion, and differentiation.^41^ Decellularization allows researchers to produce cell-derived ECM biomaterials that capture the three-dimensional (3D) complexity and metabolic factors found in native tissues by riding materials of allographic antigens and maintaining functional and structural components.^42^ Cell-derived ECM integrated in polymers are termed ECM-polymer biomaterials. ECM-polymer biomaterials combine the chemical stability of synthetic polymers and the bioactivity of ECM proteins to induce long-term cellular modulation.^43^ The use of cell-derived ECM-based biomaterial scaffolds has shown success.^44−46^

While metal-based and ECM-based composite biomaterial scaffolds hold promising results for biomedical applications, we hypothesize that a combination of metal and ECM will provide increased cell proliferation, migration, and differentiation. The combined use of PCL, Zn, and ECM will result in synergistic properties to improve the biocompatibility and bioactivity of native PCL for future regenerative medicine and wound healing applications.^47^ The goal of this study was to utilize electrospinning to create nanofiber PCL/Zn composite scaffolds, termed PZ, with varied compositions of Zn, followed by surface modification with cell-derived ECM to enhance cell proliferation, migration, and differentiation of fibroblasts.

## Experimental Section

### Materials

PCL (*M_n_* = 80,000 Da), zinc nanoparticles (Zn NPs) (40–60 nm or 0.04–0.06 μm), and Zn standard for ICP (1000 mg/L Zn in nitric acid) were purchased from Millipore Sigma (St. Louis, MO). The solvent 2,2,2-trifluoroethanol (TFE) was purchased from Alfa Aesar (Ward Hill, MA). Dulbecco’s phosphate-buffered saline (DPBS) and Dulbecco’s modified Eagle’s medium (DMEM) were obtained from Life Technologies (Grand Island, NY). For cell culture studies, an Alamar Blue assay kit, lactate dehydrogenase (LDH) assay kit, AOPI staining solution, DAPI staining solution, and ActinGreen staining solution were purchased from Thermo Fisher Scientific (Waltham, MA).

### Preparation of the PZ Nanofibers

To prepare PZ nanofiber scaffolds, PCL solution was dissolved in TFE at a concentration of 12% (w/w). Commercially available Zn NPs were added to the PCL solution under an inert atmosphere to obtain 0, 1, and 2 wt % mixtures (i.e., PZ0, PZ1, and PZ2).^48^ The solutions were subjected to constant magnetic stirring for 12 h at room temperature, followed by ultrasonication to achieve homogeneity.

The electrospinning setup and process were adopted from our earlier experiments to fabricate scaffolds.^49^ Briefly, a syringe pump (Model 78–01001, Fisher Scientific, Pittsburgh, PA), a high-voltage power supply (Model CZE100PN30, Spellman High-Voltage Electronics Corporation, Hauppauge, NY), and a collector drum were used. Approximately, 9 mL of Zn particle-loaded polymeric solution was placed in a 10 mL syringe with an attached 18 gauge diameter hypodermic needle. The syringe tip was placed 12 cm from the collector, and a 20 kV voltage supply was used to charge the solution. The flow rate was set at a flow rate of 2.5 mL/h. The solution was spun toward the rotating grounded drum wrapped with aluminum foil. The PZ0, PZ1, and PZ2 samples were placed in a chemical hood to dry overnight. Sample composition details are shown in Table 1.

**Table 1:** PZ and PZE Sample Design and Composition

fibrous scaffold	PCL-P (g)	Zinc-Z (g)	extracellular matrix -E
PZ0 (control)	1.55	0	-
PZ1	1.55	0.002	-
PZ2	1.55	0.003	-
PZ1E	1.55	as prepared	+
PZ2E	1.55	as prepared	+

### Surface Modification of the PZ Nanofibers with Cell-Derived ECM

**Figure 1:**
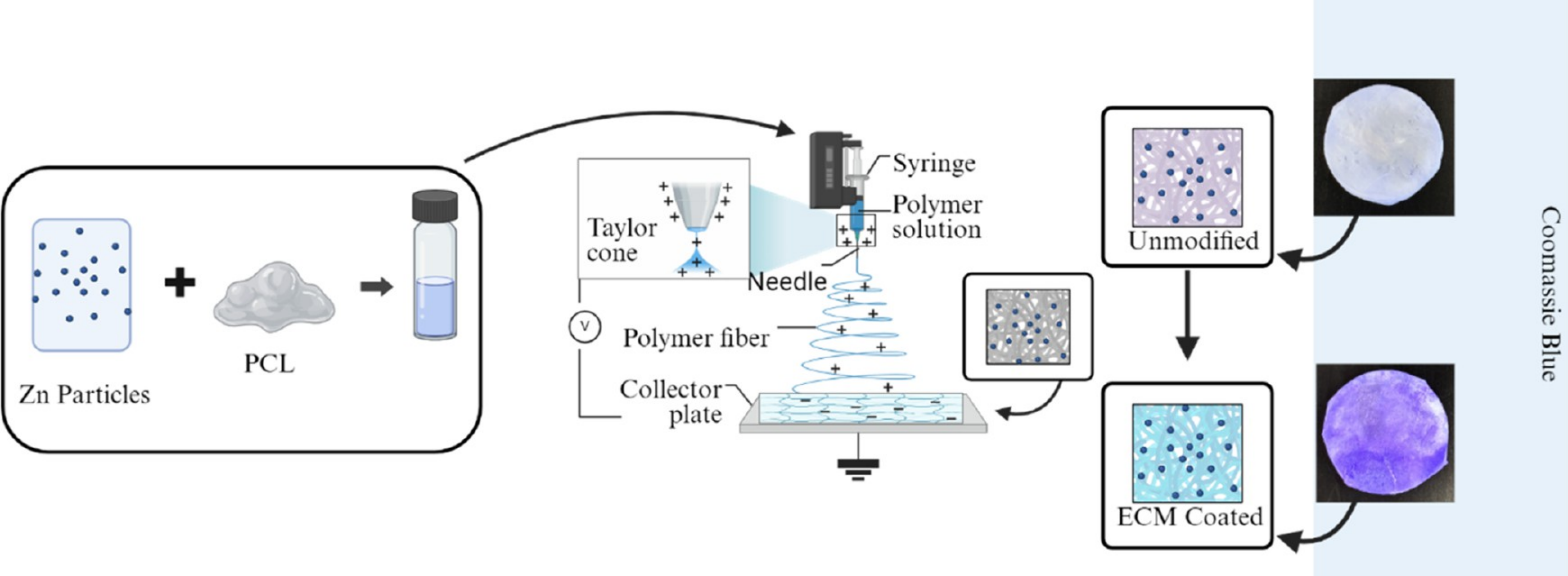
Schematic design of fiber development. (Left) The creation of composite nanofiber scaffolds of PCL and Zn using electrospinning technology, followed by surface coating of the material with fibroblast-derived ECM. (Right) Digital images of unmodified PZ fibers and PZE fibers stained with Coomassie Blue. Created in BioRender.

NIH3T3 mouse fibroblast cell lines (American Type Culture Collection, ATCC Cell Line Bank 1658, Manassas, VA) were expanded with a complete medium at 37 °C with 5% CO_2_ and a 95% humidified atmosphere. Figure 1 illustrates the schematic design of the scaffold fabrication process. The medium was replaced every third day of culture. Upon confluency, later, PZ electrospun samples were cut into squares (20 × 20 mm^2^) and welded around a 15 mm round coverglass (Carolina Biological) using TFE. Samples were sterilized with 90% ethanol under ultraviolet (UV) for 20 min with sufficient phosphate-buffered saline (PBS, Gibco; Life technologies) wash. The samples were pretreated overnight with complete media. Cells at a density of 5 × 10^4^ were seeded on the center of each scaffold and cultured in the incubator for 7 days to obtain predecellularized cell-derived PZ-ECM (PZE) samples. The cells were nourished with fresh complete media every third day. The cell-derived PZE scaffolds were decellularized by the physical freeze/thaw method according to the literature.^50^ Briefly, samples underwent freeze/thaw (−80/37 °C) treatment for three cycles followed by incubation for 60 min at 37 °C with a 1 mg/mL concentration of DNase1 (Thermo Fisher Scientific). Scaffolds were washed with PBS at each step.

### DAPI Staining

To confirm the decellularization of the cell sheet, cell-seeded PZE samples were fixed with 4% paraformaldehyde before and after decellularization. Scaffolds were then rinsed 3 times with PBS. Scaffolds were incubated with DAPI for 15 min for nuclear staining and rinsed 3 times with PBS. The scaffolds were imaged using an Olympus IX83 microscope incorporated with the Olympus cellSens Dimension software (Olympus Corporation, Shinjuku, Tokyo, Japan).

### Total Protein Quantification

The concentration of protein in each sample was assessed by using the BCA Protein Assay Kit (Thermo Fisher). Briefly, UV-sterilized PZE scaffolds were placed in 12-well plates to which 1 mL of PBS was added and then incubated at 37 °C. The total amount of protein present in PZE scaffolds was quantified using a bovine serum albumin (BSA) standard curve. To account for Zn affecting the colorimetric analysis, results from PZ samples were subtracted from PZE samples to get the total protein concentration of the added ECM.

### Fourier Transform Infrared Spectroscopy

The chemical composition of the scaffolds was analyzed via Fourier transform infrared spectrometry FTIR (Varian670 FTIR Spectrophotometer Varian,Inc.,PaloAlto,CA) at weave range of 400–6000 cm^–1^region. The amide bond peaks that signify protein detection (1600 to 1800 cm^–1^) were studied for comparison to confirm ECM surface modification.

### Surface Morphology

The micromorphology of PZ fibers and surface-modified PZE fibers was observed via scanning electron microscopy (SEM, Zeiss Auriga series, Oberkochen, Germany). For the morphological evaluation, fibers were cut into small pieces, attached to copper tape, and sputter coated with gold using a coating system (Leica EM ACE200, IL) for 30 s (coating depth = 5 nm) at 15 mA. The fiber size distribution of PZ scaffolds was analyzed using SEM images and ImageJ software (NIH, Bethesda, MD). The diameter was converted to pixels with the help of a scale bar (10 μm). Fifty individual fibers of PZ and PZE samples from each group (*n* = 3) of SEM images were measured in pixels. The average size and standard deviation were calculated based on the ImageJ data.

### Mechanical Property

The mechanical properties of the nanofiber samples were analyzed using a TA.XT Plus Texture Analyzer (Hamilton, MA). A customized template was constructed out of cardstock (25 mm × 18 mm) to hold the sample in place and ensure uniformity in loading. A sample (15 mm × 8 mm) was firmly affixed to the template with double-sided tape at both ends (*n* = 3) before sample testing. A digital micrometer was used to measure the thickness of the samples. Before testing, both sides of the template were cut as the affixed sample was placed in between the pneumatic jaw gripping plates. According to prior work, the sample was stretched until failure with 500 N load cells and a set displacement of 3 mm/min.^51^ After each run, a stress (MPa) versus strain (mm/mm) curve was generated. Stress–strain curves were plotted in OriginPro, and Young’s modulus (YM) was determined.

### Wetting Property

The wettability of the scaffolds was determined through the static contact angle measurement using the sessile drop method as shown in previous works (Rame Hart model 260 goniometer/tensiometer) at RT.^52^ The setup for this test included a vertical clamp where the syringe containing deionized water was secured for the experiment. A highly focused light source is placed at one end, and a camera is placed on the other end. The computer is connected to a computer system, which is used to capture the images. The optical image of each nanofiber scaffold absorbing water droplets was taken at the 10 s time point after the deposition on the surface of the scaffolds using DROPimage software (*n* = 3).

### Inductively Coupled Plasma Mass Spectroscopy

To measure the concentration of Zn in the scaffolds, 15 mm round samples were cut and placed in a 12-well plate before being sterilized by incubating with 95% ethanol for 20 min under UV, after which samples were washed with DI water 2 times, followed by another rinse with PBS 1 × 1 time. 1 mL of complete media DMEM + 10% fetal bovine serum (FBS) + 1% antibiotics was added to each sample and incubated at 37 °C and 5% CO^2^ atmosphere overnight. The next day, the complete medium was removed, and samples were set out to dry overnight. The samples were weighed before they underwent digestion processing with concentrated nitric acid (67–70%, Fisher Scientific) and hydrofluoric acid (48–51%, VWR Chemicals). All of the samples were then analyzed by using the Optima 8300 ICP OES instrument.

### Biological Studies of PZ and PZE Scaffolds

#### Cell Viability Assay

NIH3T3 viability on the scaffolds was evaluated using an Alamar Blue colorimetric assay. Briefly, NIH3T3s (3 × 10^4^) were cultured on scaffolds in 12-well plates for up to 3 days in a humidified atmosphere with 5% CO_2_ at 37 °C (*n* = 3). At days 1, 2, and 3 each sample was incubated for 4 h with 10% Alamar Blue reagent and culture media at 37 °C. Assay solutions were transferred to 96-well plates to measure fluorescence (530 nm excitation and 590 nm emission) by a microplate reader (CLARIOstar Plus, BMG LABTECH Inc., Cary, NC).

#### Cell Proliferation Study

The proliferation of the cells on the scaffolds was examined with AOPI (PerkinElmer LLC Via AOPI Staining Solution; Fisher Scientific) following the company protocol. The live cells stained in green and the dead cells stained in red were visualized using an Olympus IX83 microscope incorporated with the Olympus cellSens Dimension software (Olympus Corporation, Shinjuku, Tokyo, Japan).

#### Cell Morphology Study

The morphology of the NIH3T3 cells on the scaffolds was visualized under a fluorescence microscope in cells cultured for 3 days. The cells were seeded at a density of 1.0 × 10^4^ cells/well. After 3 days, the cells were washed 3 times with PBS before fixing with 4% paraformaldehyde (PFA, Thermo Fisher Scientific) solution and permeabilized in 0.2% Triton (X-100) (Thermo Fisher Scientific) for 10 min at RT. After being washed with PBS, the cells were blocked with 1% bovine serum albumin (BSA) for 30 min. The cells were then stained with DAPI (4′6-diamidino-2-phenylindole dihydrochloride; Invitrogen, Thermo Fisher Scientific) for nuclei (5 min) and ActinRed Readyprobes reagent (Invitrogen, Thermo Fisher Scientific) for the cytoplasm (20 min) at RT covered with aluminum foil. After washing 3 times with PBS, fluorescent images were captured in a dark room using an Olympus IX83 microscope incorporated with the Olympus cellSens Dimension software (Olympus Corporation, Shinjuku, Tokyo, Japan).

#### Cytotoxicity Assay

Cytotoxicity of the PZE scaffolds was evaluated by using the Pierce LDH assay kit (Thermo Fisher). NIH3T3s (3 × 10^4^) were cultured on scaffolds in a 12-well plate in a humidified atmosphere with 5% CO2 at 37 °C (*n* = 3). At days 1, 2, and 3, 50 μL of sample media was collected and stored at −80 °C for further analysis. Briefly, 50 μL of collected media was transferred to a 96-well plate and mixed with 50 μL of reaction mixture. The plate was covered with aluminum foil and incubated at RT for 30 min. Stop solution was added to each well to stop the reaction, and the absorbance of the samples was measured at 490 and 680 nm by a microplate reader (CLARIOstar Plus, BMG LABTECH Inc., Cary, NC).

#### Cell Scratch and Would Closure Evaluation

A scratch assay was performed to study the effect of the PZ and PZE scaffolds on the cell migration activities. The spreading and migration ability of NIH3T3 fibroblasts was assessed using a scratch model. The cells were seeded into a 24-well plate at a concentration of 5 × 10^4^ cells/well and cultured in complete media until 90% confluent. Then, using a sterile 200 μL plastic pipet tip, a linear scratch was generated on the cell monolayer. Cellular debris was removed by washing with PBS. Simultaneously, all fibers (PZ0, PZ1, PZ1E, PZ2, and PZ2E) were submerged in complete media for 24 h and stored as conditioned media (*n* = 3). Complete media was removed from the 24-well plate and replaced with conditioned media. Images of each scratch assay sample were taken at time points 0, 6,12, and 24 h using Life Technologies EVOS FL inverted microscope. ImageJ software was used to calculate the closure rate of the scratch assay pictures.
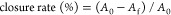
*A*_0_ and *A*_f_ represent the area at 0 h and the final 24 h time point, respectively.

#### Immunohistochemical Staining

The identification of differentiated fibroblasts was performed using immunohistochemical staining to visualize α- smooth muscle actin (α-SMA). Briefly, scaffolds were placed into 12-well plates and sterilized according to the previously described protocol. NIH3T3 cells were seeded directly on the scaffolds at a density of 1.0 × 10^4^ cells and allowed to expand in a 37 °C incubator for 14 days. Media was changed every third day. The scaffolds were fixed in 4% paraformaldehyde and treated with DPBS. The fixed cells were incubated with rabbit monoclonal antibodies to α-SMA antibodies (Abcam). Samples were then incubated with fluorescent-conjugated secondary antibodies goat antirabbit. The labeled cells were washed with DPBS and imaged using an Olympus IX83 microscope incorporated with the Olympus cellSens Dimension software (Olympus Corporation, Shinjuku, Tokyo, Japan).

#### Statistical Analysis

Statistical analysis was performed using OriginPro 2024 (Origin Lab, Northampton, MA). The mean ± standard deviation (SD) was used to represent average values. Post hoc Tukey’s test ANOVA for multiple comparisons was used to explore the differences between means. Statistical differences were considered to be statistically significant when *p* < 0.05.

## Results and Discussion

### Characterization of PZE Scaffolds

**Figure 2:**
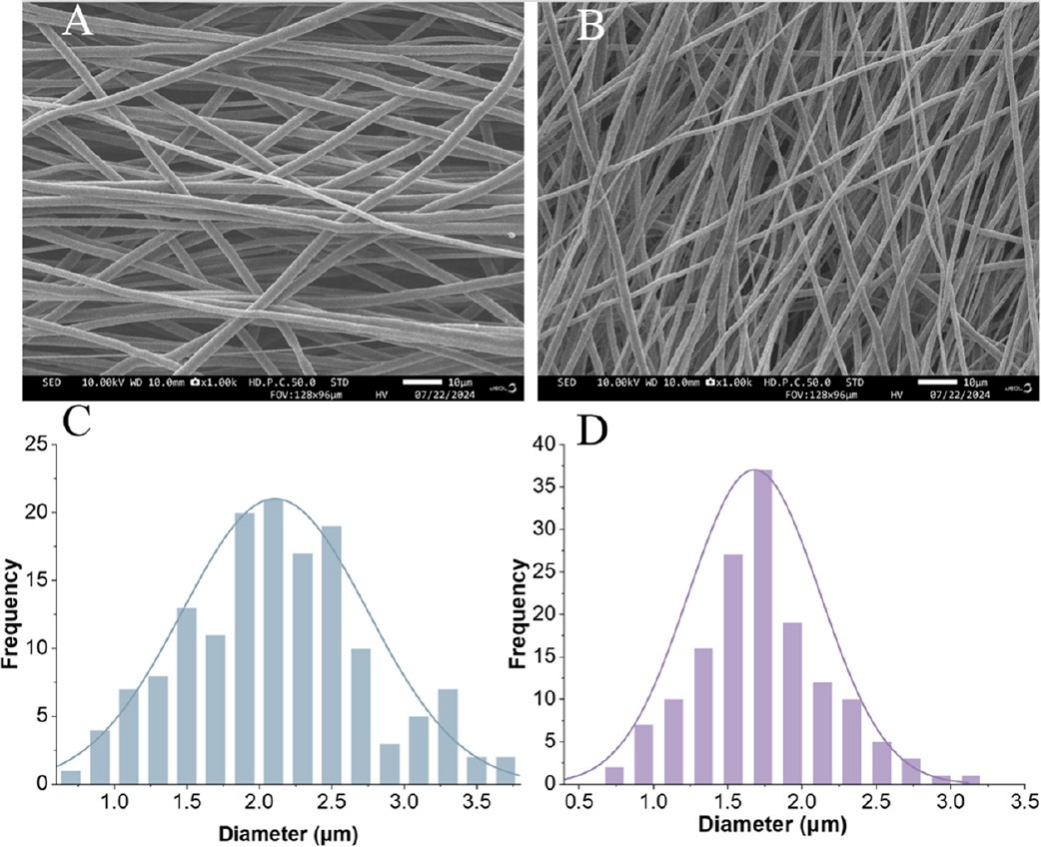
Surface morphology and fiber diameter analysis of PZ fibers. (A) SEM image of PZ1 fibers. (B) SEM image of PZ2 fibers. Scale bar 10 μm (*n* = 3). (C) Histograms showing fiber diameter distribution frequency in PZ1 fibers, *n* = 50. (D) Histograms showing fiber diameter distribution frequency in PZ2 fibers, *n* = 50.

Before ECM coating, PZ nanofibers were prepared via electrospinning using a mixture of PCL and Zn particles dispersed in TFE solvent (Figure 1). SEM images reveal that as Zn was added, the average fiber diameter decreased from 2.11 ± 0.5 to 1.68 ± 0.3 for PZ1 and PZ2 (Figure 2). This reduction might be due to the distribution of Zn particles which made the electrospinning solution more electronically conductive, causing thinner fibers. Both PZ1 and PZ2 samples contain Zn, which contributes to the overall metal content. The higher concentration of Zn in the PZ2 samples leads to an increased charge, facilitating a greater stretching effect from the nozzle to the collector during the electrospinning process. This enhances stretch and can result in the formation of thinner fibers.^53,54^

We chose NIH3T3 fibroblasts to modify PZ fibers because NIH3T3 fibroblasts serve as a model system for cell cycle studies. They are known to have primary cilia that play an essential role in determining the direction of cell migration.^55,56^ Fibroblasts are also the main producers of ECM in animal tissues, which makes NIH3T3 cells ideal for this study, which aimed to produce ECM-coated nanofiber scaffolds.^57^ Biologically, Zn is involved in cell catalytic, structural, and regulatory functions, including serving as a cofactor for over 300 enzymes.^35^

**Figure 3:**
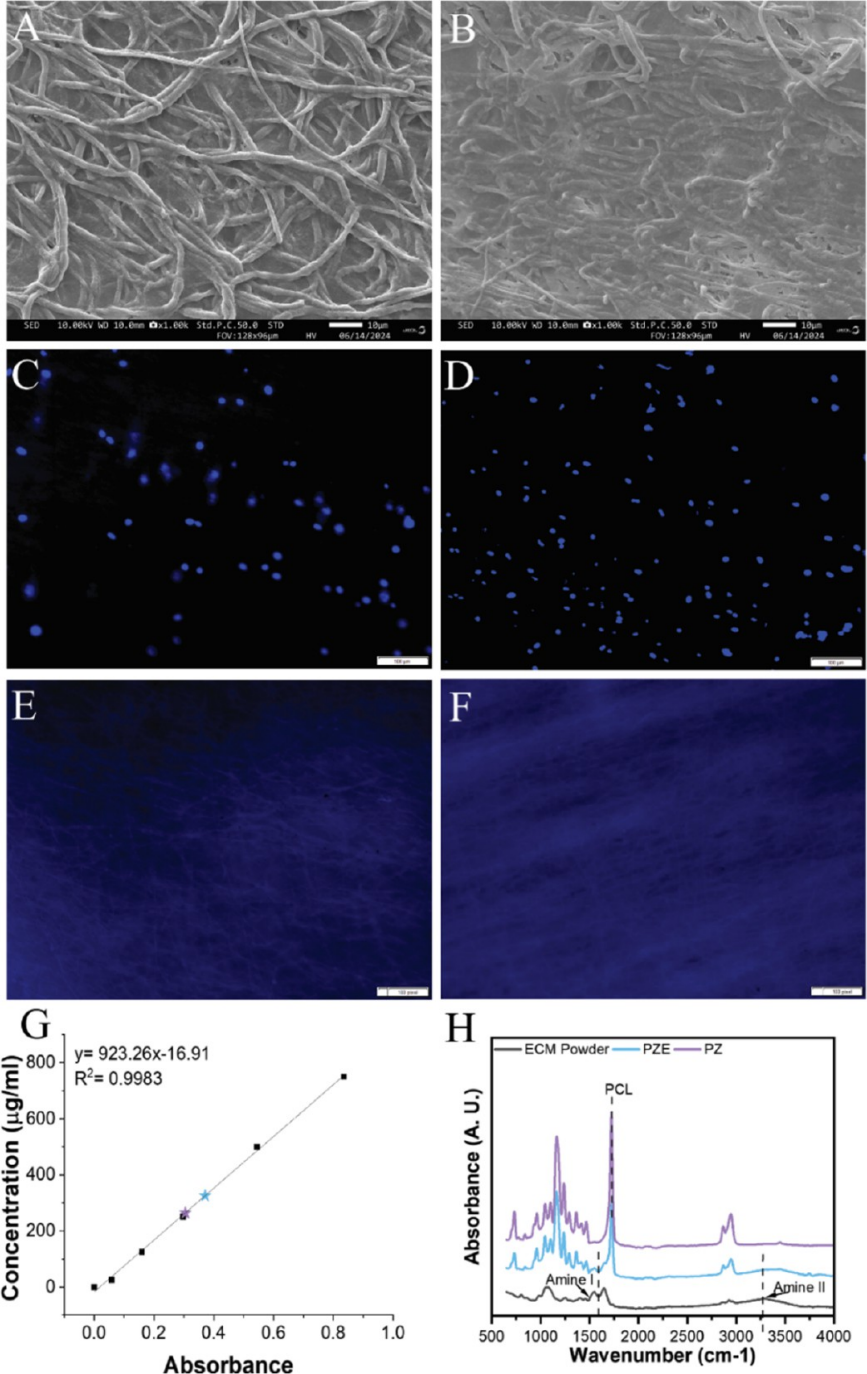
Analysis of ECM protein on PZE fibers. (A) SEM image of PZ1E fibers. (B) SEM images of PZ2E fibers. Scale bar 10 μm. (C) DAPI stained PZ1E fibers before decellularization. (D) DAPI stained PZ2E fibers before decellularization. (E) DAPI stained PZ1E fibers after decellularization. (F) DAPI stained PZ2E fibers after decellularization. Scale bar 100 pixels. (G) The total protein concentration on PZ1E (purple) and PZ2E (blue) fibers relative to the BCA standard curve. (H) FTIR spectra confirming the presence of ECM proteins.

New fibers, termed PZE, were analyzed for decellularization and protein deposition. Cell-based ECM deposition with freeze–thaw decellularization was used to modify PZ fibers. Both PZ1E and PZ2E nanofiber scaffolds appear to have a net-like coating on the surface (Figure 3A,B).^58−60^ The ECM coating was denser on PZ2E fibers compared to PZ1E fibers. We assume that this increase in ECM protein is because Zn leads to increased cell growth causing more ECM production.^34,61^

DAPI, a blue fluorescent DNA stain, was used to confirm that decellularization removed the nuclear DNA content from the PZE scaffolds. Figure 3C–F shows images of the nuclear DNA content on PZ1E and PZ2E fibers before and after decellularization. We observed that our decellularization process removed the cellular genetic content from the cell-seeded nanofibers. Decellularized biomaterials have been studied by researchers because not only does the technique eliminate cells, but it also eliminates cellular components like DNA, which is important to prevent any adverse immune responses.^62^ Consistent with the literature, results confirm the complete removal of cells with minimal elimination of the ECM from the surface using freeze–thaw decellularization.^63^

To confirm the presence of ECM proteins on PZE fibers after decellularization, a bicinchoninic acid assay (BCA) was conducted (Figure 3G). PZ1E fibers showed approximately 235 (μg/mL) of protein after being submerged in PBS for 24 h. In contrast, the PZ2E fibers showed an increase in protein content with a concentration of approximately 328 (μg/mL). Findings further verify that Zn increases ECM deposition.

To further confirm the presence of ECM proteins, the existence of amine groups was confirmed by spectroscopy using FTIR spectroscopy (Figure 3H). An amine I peak at 1654 cm^–1^ confirmed the presence of collagen type I in the PZE membranes.^64^ Collagen type I is the most prominent ECM component in cell-derived ECM.^65^ An additional peak at 3450 cm^–1^ is a characteristic found in amine backbones indicating the presence of additional ECM proteins.^66^ The peaks that are associated with amide bonds were absent in the PZ fibers but present in the ECM powder and PZE scaffolds. Decellularized cell-derived ECM has been shown to stimulate cellular proliferation, modulate differentiation, and reduce possible chromosomal abnormalities making it an ideal bioactive material.^67,68^ Therefore, using cell-derived ECM to enhance PZ nanofibers’ performance is a feasible approach for synthesizing a novel biomaterial that enhances cell proliferation, migration, and differentiation.

### Mechanical Properties of Scaffolds

**Figure 4:**
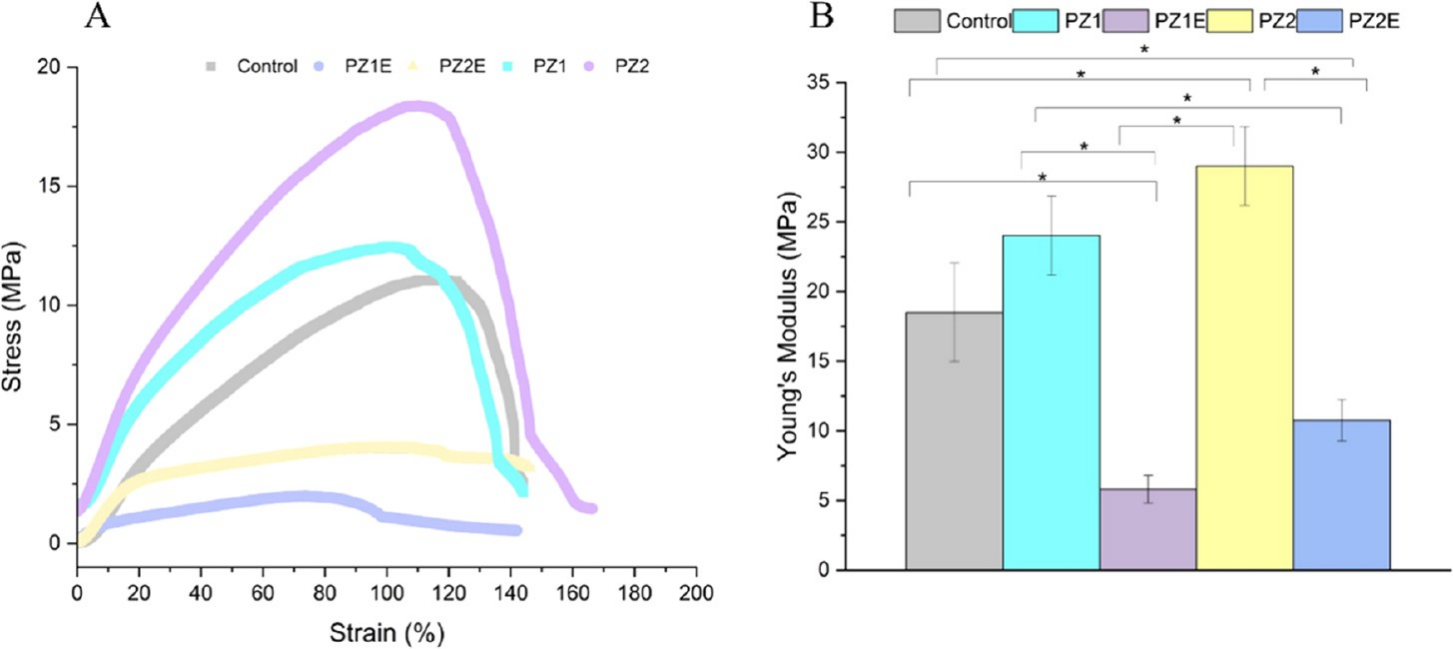
Analysis of the mechanical properties of various nanofiber scaffolds. (A) Representative tensile stress–strain curves for the nanofiber scaffolds. (B) Young’s modulus of the nanofiber scaffolds. Statistical significance was determined using the one-way ANOVA post hoc Tukey method and data were expressed as mean ± S.D., *n* = 3 (where *p < 0.05).

The tensile mechanical properties of the various nanofiber scaffolds are expressed in stress vs strain curves (Figure 4A), and Young’s modulus (Figure 4B). The mechanical properties of a scaffold play an important role in the growth of cells and the regeneration of tissues.^69^ The PZ1 and PZ2 scaffolds showed higher tensile strength and higher Young’s modulus than PZ0, PZ1E, and PZ2E scaffolds. The Young’s modulus was 18.5 ± 3.53 MPa, 24 ± 2.82 MPa, 5.8 ± 0.89 MPa, 29 ± 2.82 MPa, and 10.75 ± 1.48 MPa for PZ0, PZ1, PZ1E, PZ2, and PZ2E scaffolds, respectively. There was also an observed decrease in the ultimate tensile strength as Zn and ECM protein was added to the PCL control. One cause could be the distribution of Zn particles within the fibers. Incorporating metals to polymers is known to increase the tensile strength and Young’s modulus, as the metals contribute to the local density, stiffness, and toughness of the composite material.^70^ We also observed that the addition of ECM proteins onto the nanofiber surface resulted in a decrease in both Young’s modulus and tensile strength, a trend that has been reported in similar studies.^71−73^ Notably, human skin has a Young’s modulus that ranges from 4.6–20 MPa.^74^ While the addition of Zn alone increases the Young’s modulus of the fibers above this range, the presence of ECM helps to counteract this effect, bringing the Young’s modulus back in line with that of native skin. Therefore, if the PZE scaffolds were used for skin tissue regeneration applications, the Young’s modulus would remain in the range of native tissue.

### Wettability of the PZ and PZE Scaffolds

**Figure 5:**
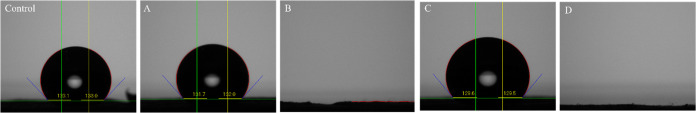
Images showing the contact angle measurement of the PZ and PZE samples after 10 s. Where control = PZ0, A = PZ1, B = PZ1E, C = PZ2, D = PZ2E; *n* = 3.

The hydrophilicity of the PZ and PZE scaffolds was analyzed after the contact angle was measured. The average contact angle measurements after 10 s for the Control, PZ1, PZ1E, PZ2, and PZ2E samples were 133.15 ± 0.07, 131.35 ± 0.21, 0, 128.55 ± 0.07, 0°, respectively (Figure 5). These values indicate that PCL is extremely hydrophobic. The addition of Zn significantly decreased the contact angle and increased the hydrophilicity (*p* < 0.05). This is due to the hydrophilic nature of Zn ions.^75^ When the ECM samples were analyzed, the water droplet was completely absorbed into the fibers after the 10 s end point. Similar studies show that surface modification with ECM protein increases the hydrophilicity of scaffolds.^76,77^ The coupled interactions between Zn and ECM proteins with the PCL enhance the scaffold’s wettability and hydrophilic properties, which are essential for promoting cell proliferation and migration.

### Concentration in PZE Scaffolds Zinc

Zn is an essential micronutrient that is found in the human body. ICP was performed to confirm the presence of trace amounts of Zn in the samples. The samples were subjected to cell culture sterilization protocols before being dried and analyzed. The amount of Zn (wt %) in samples was 0, 0.02 ± 0.007, 0.14 ± 0.021, 0.01 ± 0, and 0.07 ± 0.021 for control, PZ1, PZ2, PZ1E, and PZ2E fibers (See Supporting Information: Table S1). The Zn in PZ1E and PZ2E decreased by 50%. The presence of DMEM, which includes amino acids and vitamins, can lead to a pH drift, which can increase the degradation rate of PCL, which we believe contributed to the loss of Zn in the media of PZE scaffolds during the 7-day ECM deposition protocol.^78^ Nonetheless, according to the literature, the total amount of Zn in women is 1.5 and 2.5 g in men.^79^ While most Zn is found in bone and skeletal muscle, 5% of the micronutrient is found in skin reserves.^32^ We believe the presence of trace amounts of Zn in PZ2E nanofibers contributed to increased ECM deposition as shown in Figure 3A,B. Studies show that Zn supplementation increases collagen production activity, which is the most abundant protein found in ECM.^80,81^

### Biocompatibility of PZE Scaffold

#### Live/Dead Staining

**Figure 6:**
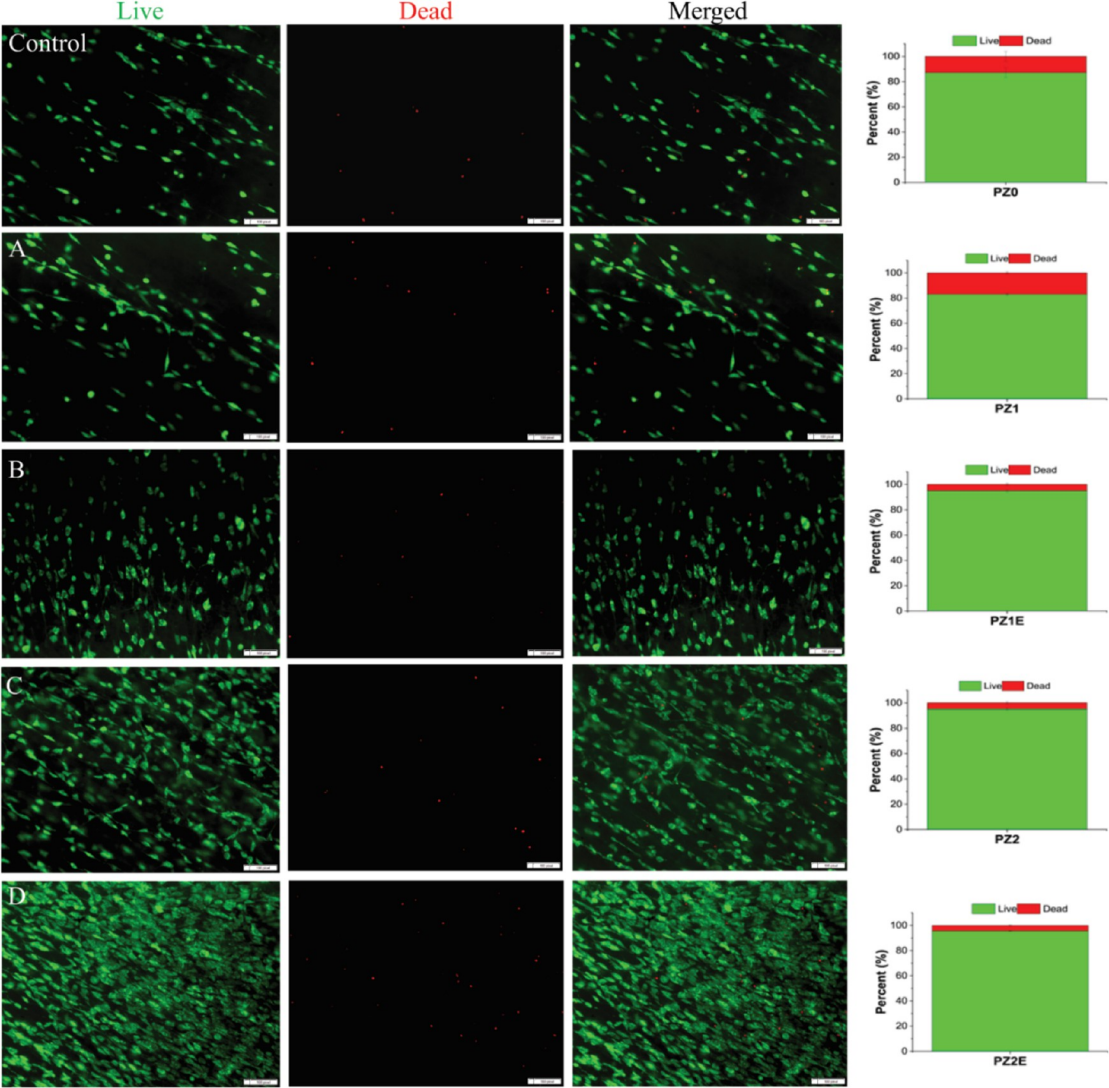
In vitro analysis of live and dead cells. (Left) Fluorescence microscopy images represent live and dead NIH3T3 fibroblasts cultured on the PZ and PZE scaffolds for 3 days utilizing acridine orange/propidium iodide (AOPI) dye. Where A = PZ1, B = PZ1E, C = PZ2, and D = PZ2E (Right). Histograms show the counted percentage of live and dead cells in the corresponding fluorescence images using ImageJ software. Data are expressed as mean ± S.D, *n* = 3. Scale bar = 100 μm.

To distinguish between live and apoptotic cells seeded on PZ1, PZ1E, PZ2, and PZ2E nanofibers, we performed AOPI live/dead staining (Figure 6). The live/dead assay was used to visualize and quantify the distribution of living and dead cells after 3 days on the scaffolds. Live cells are stained green, and dead cells with a compromised membrane are stained red. After day 3 all samples exhibited many live cells and few dead cells. The cells that were seeded on the control scaffolds appeared green but had a lower cell density compared with when Zn and/or ECM were added to the fibers. Some of the cells indicated apoptosis, but the majority of the cells remained healthy and viable. In a bone regeneration study, researchers found that in a range of 0–2 wt % Zn, the bone promotion effect of PCL/Zn scaffolds increases with increasing Zn content. However, when the content of Zn was increased to 3 wt % the bone-promoting effects were decreased.^82^ Consistent with the literature, the number of cells on our PZ2 scaffolds was higher than those on the PZ1 scaffolds. Similarly, there were more green cells in the PZ2E scaffolds compared with the PZ1E scaffolds. Overall, both PZE samples, regardless of Zn concentration, had higher viability than the corresponding PZ samples. Overall, we can conclude that none of the scaffolds had obvious cytotoxicity, and the inclusion of Zn and an ECM coating provides a better environment for the cells to remain viable and proliferate.

#### Cell Morphology

**Figure 7:**
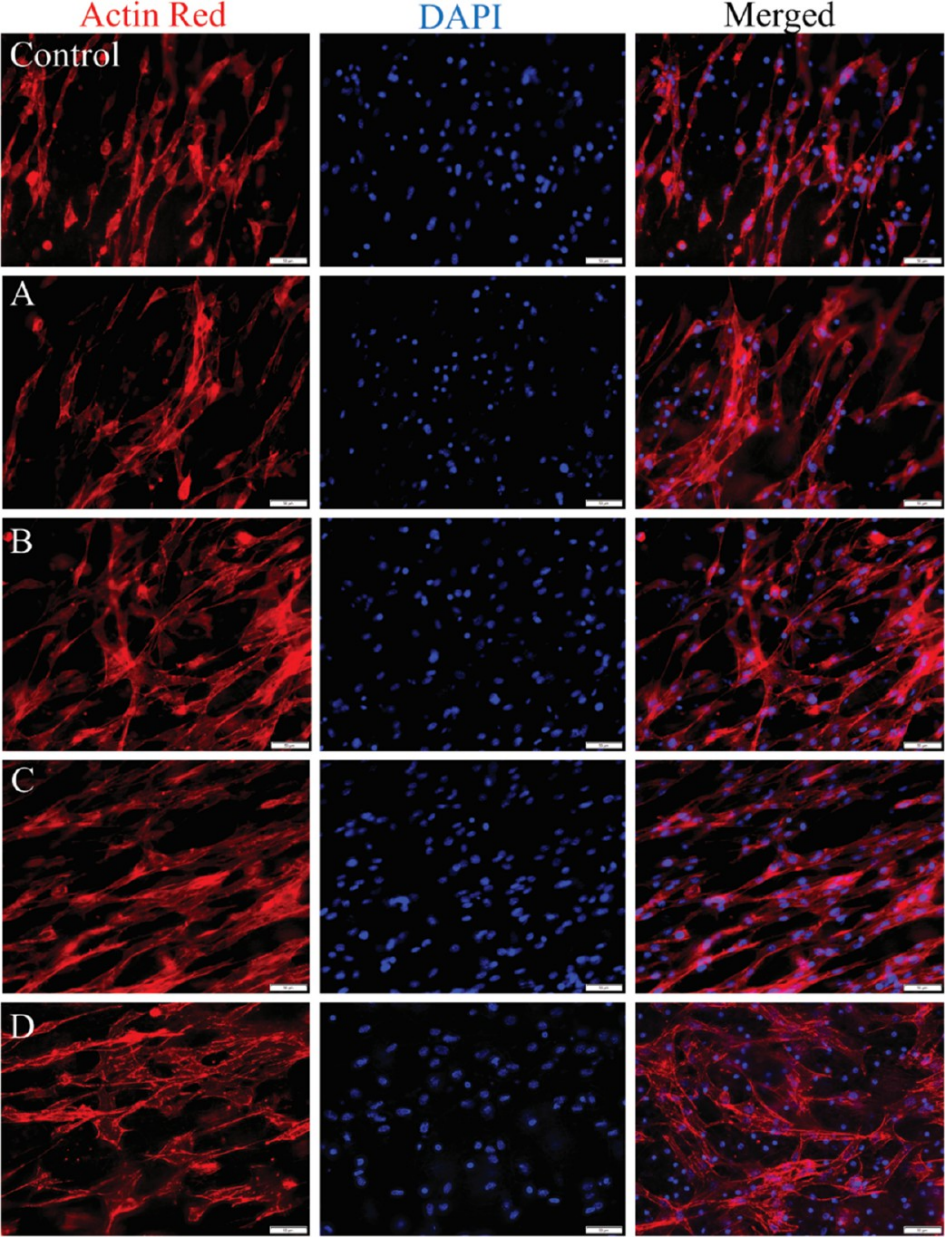
In vitro analysis of the cytoskeleton morphology. Fluorescence microscopy images represents cytoskeleton and nucleus of NIH3T3 fibroblasts cultured on the PZ and PZE scaffolds for 3 days utilizing ActinRed and DAPI dye. Where A = PZ1, B = PZ1E, C = PZ2, and D = PZ2E. Scale bar 50 μm.

Cell morphology staining using fluorescence microscopy revealed the cell shape and adhesion behavior of NIH3T3 cells. When NIH3T3 cells exhibit a flat structure and polygonal shape. The morphological response of fibroblasts was analyzed to characterize the behaviors between NIH3T3 fibroblasts and the PZ1, PZ1E, PZ2, and PZ2E scaffolds (Figure 7). Cell staining with ActinRed was conducted for 3 days, following direct cell seeding on the scaffold surface. Actin stress bundles were identified as bright red regions. DAPI, a nuclear stain, was identified as the blue regions. An interconnected morphology of cells was observed on all scaffolds, which includes the characteristic spindle-like morphology typically seen in NIH3T3 fibroblasts.^83^ More specifically, the ActinRed stain revealed a flattened polygonal shape with a well-distributed dendrite architecture. Nuclei were present throughout the sample both at the surface and interporous level, which is shown by DAPI.

**Figure 8:**
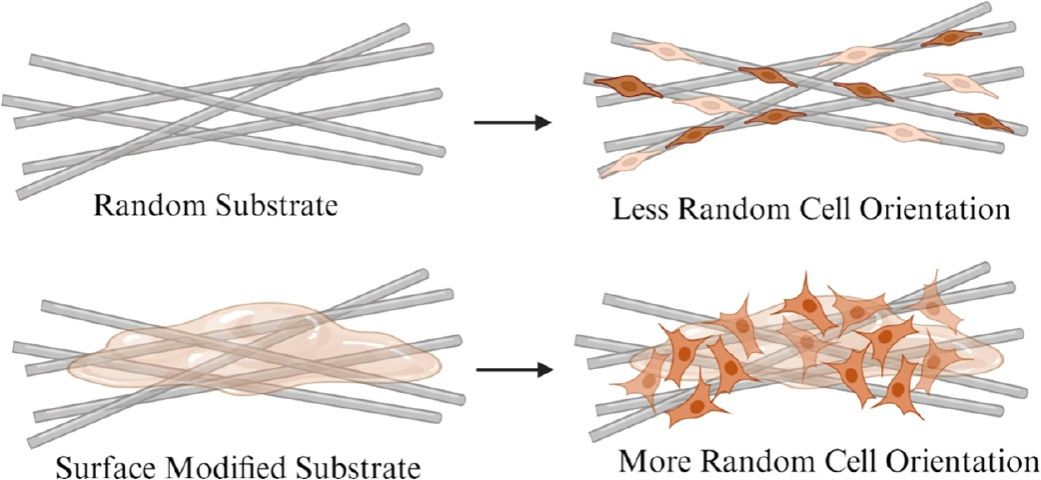
Illustration of the influence of fiber orientation and cell orientation on random- and surface-modified substrates. Created in BioRender.

It has been shown that NIH3T3 cell orientation is determined by the orientation of fibers where fiber orientation leads to a directional growth of cells along the fibers.^84,85^ Here, we observe that the control, PZ1, and PZ2 scaffolds lead to a more aligned orientation of the cells. However, cells appear to have more of a random orientation when seeded on PZ1E and PZ2E scaffolds (Figure 8). It has also been reported that cells cultured on fibers tend to change their orientation and elongate in the direction of fibers while fibroblasts cultured on flat and random surfaces do not show this behavior.^86^ Bashur et al. observed that the projection area of NIH3T3 fibroblasts increased with increasing fiber diameter and degree orientation when seeded on PLGA fibers.^87^ These results indicate that while surface-modified PZE scaffolds do not impact cell morphology, they do cause the cells to grow in a slightly less aligned orientation because of the ECM coating shown in the SEM images (Figure 3A,B).

#### Cell Viability and Proliferation

**Figure 9:**
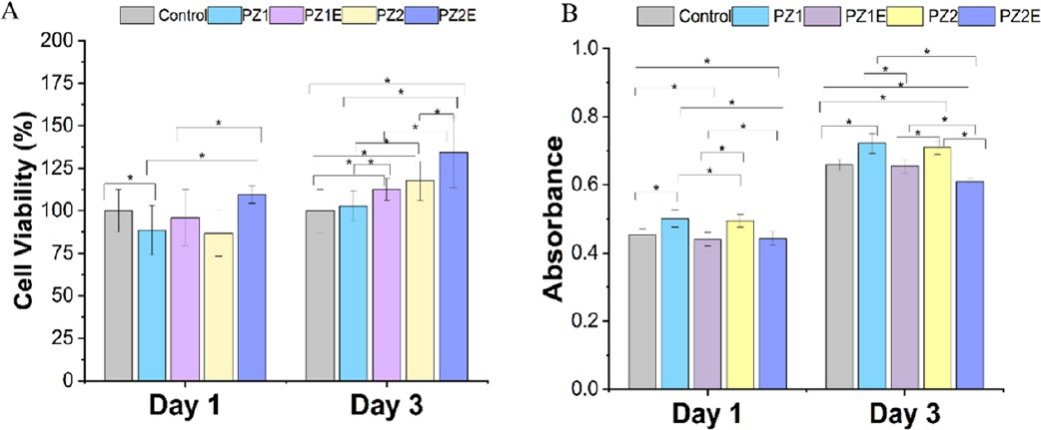
In vitro cell viability and cytotoxicity. (A) Percentage of cell viability of cells cultured directly on PZ and PZE scaffolds using colorimetric Alamar Blue assay at days 1 and 3. (B) Colorimetric LDH assay to measure the cytotoxic effect of cells directly cultured on PZ and PZE fibers at 1 and 3. Statistical significance was determined using the one-way ANOVA post hoc Tukey method and data were expressed as mean ± S.D., *n* = 3 (where *p < 0.05).

Fibroblasts produce ECM, which functions to support new cellular growth.^88^ Here, PZE scaffolds were designed to improve cell proliferation using fibroblast-derived ECM. To measure the cell proliferation and viability of NIH3T3 fibroblasts seeded on PZE scaffolds, cells were cultured directly on scaffolds for 3 days (Figure 9A). Quantitative analysis of cell proliferation was done using the Alamar blue assay, which is a trusted reagent for cell viability and cell proliferation measurements.^89^ Results showed that by day 3, fibroblasts grown on the PZE1 scaffolds showed significantly more growth than those on the PZ1 scaffolds. Similarly, on day 3, cells on PZ2E showed significantly more growth than cells seeded on PZ2 scaffolds. All of the scaffolds had significantly more growth compared to the control on day 3 except for cells seeded on PZ1. Cells seeded on PZ1E scaffolds had 12% more proliferation than the control and 10% more cellular proliferation than PZ1 scaffolds. While cells seeded on PZ2E samples had 34% more viability than the control samples and 17% more proliferation than PZ2 samples. We attribute this finding to the synergistic effects of Zn and ECM modification contributing to higher viability and cell proliferation in PZE samples.^90,91^

The acute cytotoxicity of PZE scaffolds was assessed by evaluating fibroblast response using the LDH assay. The LDH assay assesses the level of plasma membrane damage.^92^ On day 3, the LDH level in the control groups and all of the fiber matrices were evaluated. PZE scaffolds exerted lower cytotoxicity on NIH3T3 scaffolds compared to the control (Figure 9B). PZ samples had a higher cytotoxicity compared with their PZE correspondents. Similarly, Wang et al. found that there was no significant cytotoxicity of ECM-coated fibers when evaluating the effects of ECM cell sheets and bone marrow mesenchymal stromal cell behavior.^93^ We believe that the ECM surface modification, as well as the loss of Zn during the decellularization process, provided a slight level of cytotoxicity protection caused by adverse Zn toxicity.

#### Scratch Assay

**Figure 10:**
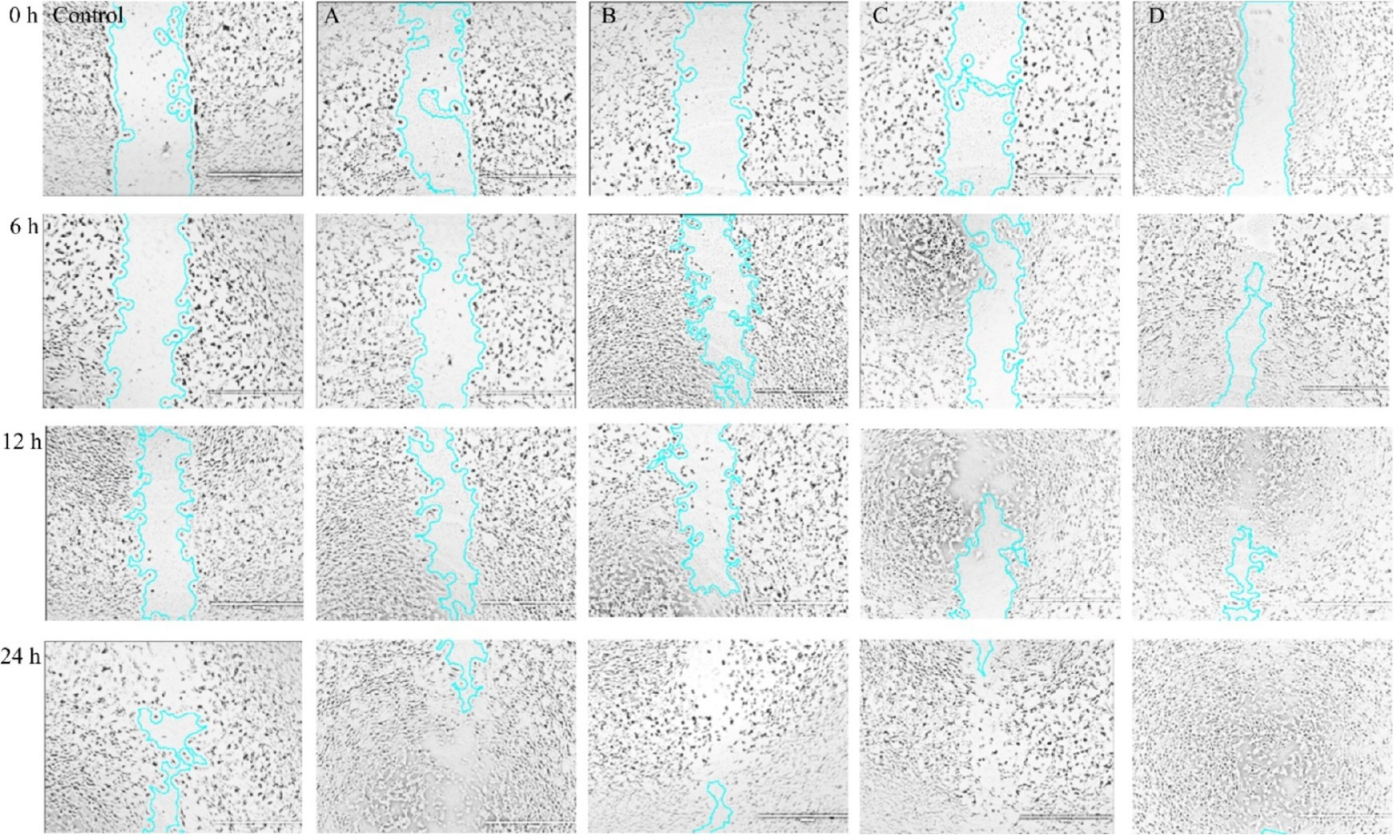
In vitro scratching effect of PZ and PZE on fibroblast migration. Cell motility and migration were observed at 0, 6, 12, and 24 h, respectively, after being exposed to conditioned media where A = PZ1, B = PZ1E, C = PZ2, and D = PZ2E. Images are acquired by optical microscopy. Scale bar 1000 μm.

Studying the migration of cells in a confluent monolayer using controlled in vitro conditions allows researchers to stimulate and explore the actions of cell migration.^94,95^ We used the scratch assay to examine the effects of the PZ and PZE scaffolds on the migration and invasion of NIH3T3 fibroblast cells (Figure 10).

**Figure 11:**
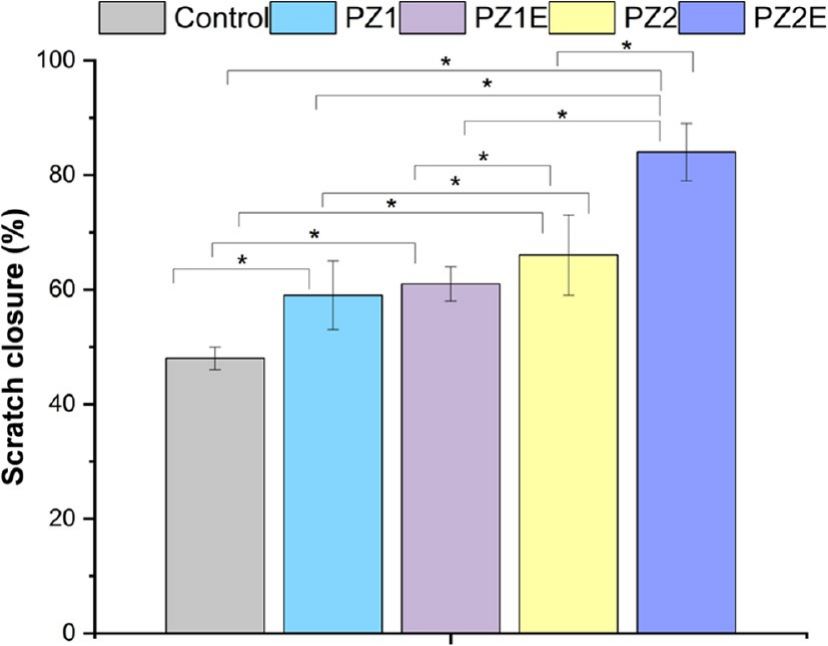
Relative cell scratch closure area percentage. Quantification of cell migration after 24 h. Statistical significance was determined using the one-way ANOVA post hoc Tukey method and data were expressed as mean ± S.D., *n* = 3 (where *p < 0.05).

The scratch assay allows for quantification of the rate at which the scratch closes without using skin equivalents. To investigate the percentage of scratch closure, we used ImageJ software to quantify the closure rate of cells by using images taken during the scratch assay. The closure rate was demonstrated by calculating area differences at 0 and 24 h. The migration of cells seeded on PZ2E (80%) was higher as compared to control (43%), PZ1 (55%), PZ1E (57%), and PZ2 (65%) (Figure 11).

Soluble Zn ions and ECM components released from the nanofiber scaffolds into the media may enhance cell migration.^96,97^ The release of these soluble factors can affect the cell behavior. Studies report that Zn enhances the migratory ability of cells.^33^ Consistent with the literature, our results show that increasing the Zn concentration from 0 to 2% increases the closure rate. The addition of decellularized ECM increases the migration and infiltration of cells.^98^ Fibroblasts exposed to PZ1 fibers had the slowest closure compared with PZ1E, PZ2, and Z2E fiber-conditioned media. Whereas, PZ2E had the fastest closure rate was approximately 85% in 24 h. Overall, there was a positive influence on migration when cells were in the presence of Zn and ECM proteins.

#### Fibroblast Differentiation

Synthesis of α-SMA is a principal component of myofibroblasts, which are a central orchestrator of cellular and tissue repair. Differentiated fibroblasts form myofibroblasts that express α-SMA, which are primarily responsible for increased ECM production, cell-to-matrix adhesion, and resistance to apoptosis.^99^ In addition to being a marker for myofibroblast differentiation, α-SMA also plays in the contractile force during regeneration.^100^ McAndrews et al. found that depleting α-SMA myofibroblasts in mice lead to chronic nonhealing wounds.^101^

**Figure 12:**
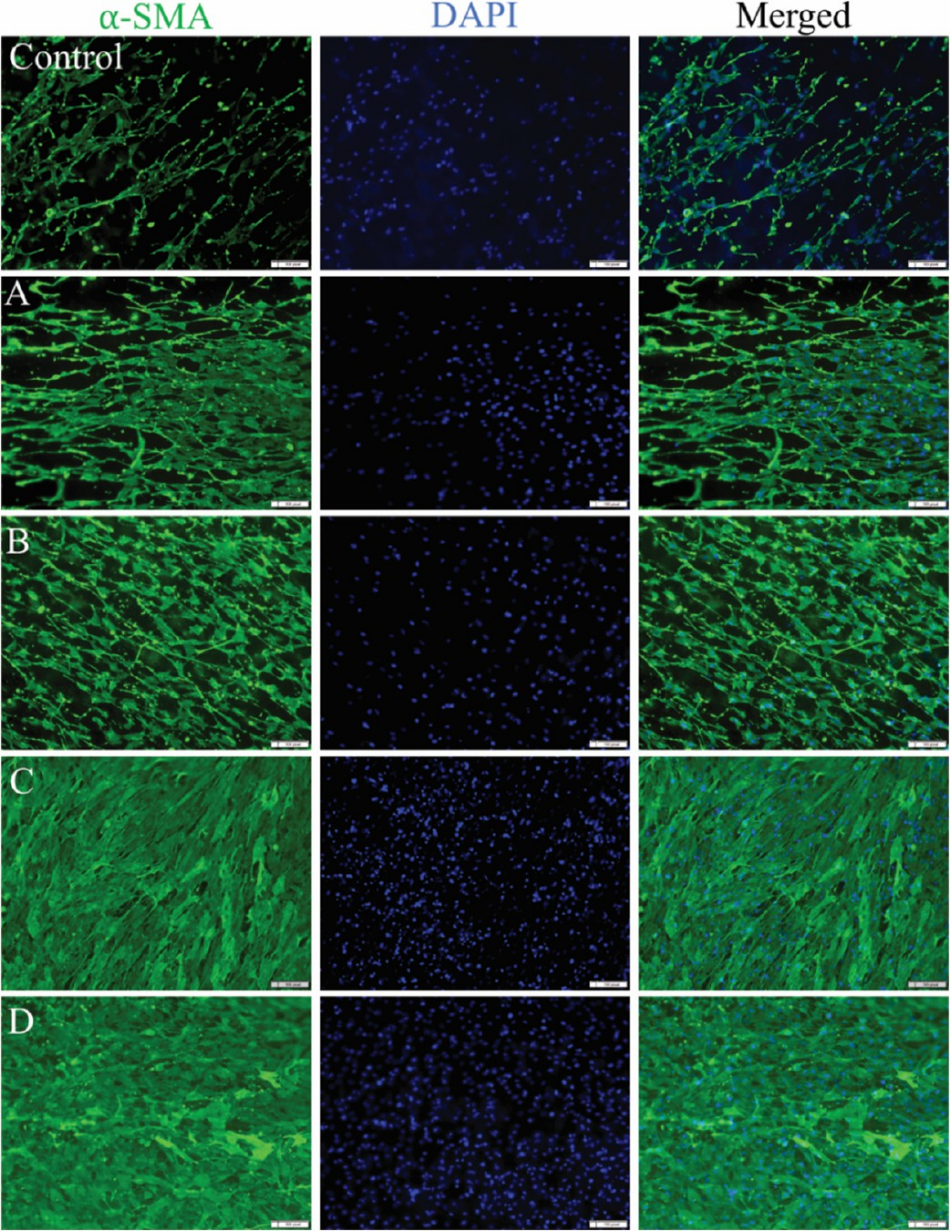
Differentiation of NIH3T3 fibroblasts into myofibroblasts. Representative fluorescence images of differentiated NIH3T3 cells. α smooth muscle actin (α-SMA), a marker of differentiation from fibroblast to myofibroblast, was used to stain NIH3T3 cells after 14 days of incubation by using immunohistochemistry, where A = PZ1, B = PZ1E, C = PZ2, and D = PZ2E. Scale bar = 1000 pixel.

**Figure 13:**
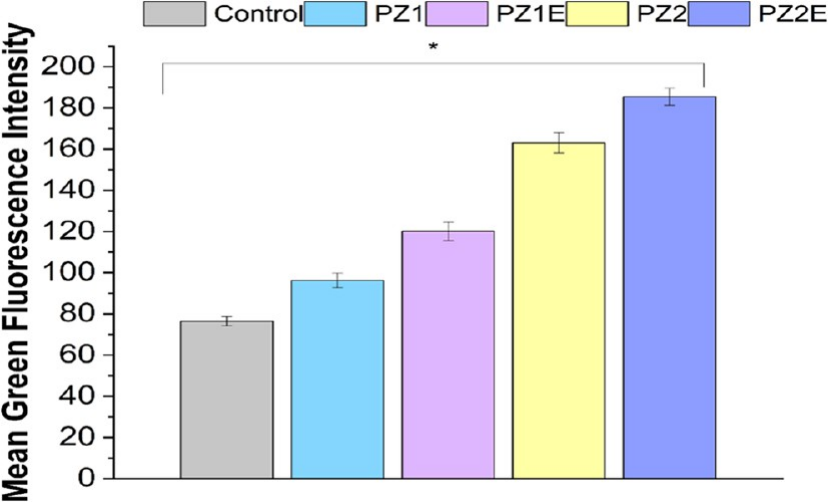
Fluorescent intensity plot of SMA expression in NIH3T3 fibroblasts grown on the PZ and PZE scaffolds. Data are expressed as mean ± S.D., *n* = 3 (where *p < 0.05).

Fluorescent images of α-SMA (green) reveal that all groups, including the control group, expressed α-SMA after 14 days (Figure 12). The images demonstrate not only an increase in cell numbers but also an enhanced expression of α-SMA, particularly following the addition of Zn and ECM proteins. This is shown by an increase in the green fluorescent intensity. The intensity, measured using ImageJ software, correlated with a higher α-SMA expression. There is a significant difference between all of the PZ and PZE scaffolds, highlighting the varied effects of each of the scaffolds on α-SMA expression. The incorporation of Zn and ECM leads to a 64% increase in α-SMA expression (Figure 13). The reduced performance of the PZ1E scaffolds compared to the PZ2 samples may be due to the higher Zn concentration, which creates a more effective environment since Zn is crucial for many cell signaling pathways.^102,103^ In the PZ1E scaffolds, the lower Zn concentration, despite the presence of ECM, may not be sufficient to activate the necessary pathways effectively, resulting in a lesser impact than that of PZ2 alone. Additionally, the PZ2 and PZ2E samples exhibited better cell proliferation and lower cytotoxicity compared to the control, PZ1, and PZ1E samples (Figure 9).

## Conclusions

In this study, decellularized PZE membranes were fabricated by NIH3T3 fibroblast cell culturing for 1 week on PZ electrospun scaffolds, followed by freeze/thaw decellularization to increase hydrophilicity and improve biocompatibility. The bioactive PZE scaffold has a promising potential for tissue regeneration. SEM images revealed that PZE scaffolds exhibited a sheet-like covering that was absent in PZ samples. Our results suggest that PZE scaffolds support cell proliferation and metabolism, as indicated by Alamar blue and live/dead experiments. The scaffolds also have limited cytotoxic effects, as shown in the LDH assay. The morphological assessment with ActinRed and DAPI revealed that the cell morphology was not compromised when NIH3T3 fibroblast cells were seeded on PZE scaffolds. The in vitro scratch assay confirmed that PZ2E fibers contributed to increased cell migration. Staining for α-SMA shows an increased expression in PZ2E scaffolds, which signifies the presence of myofibroblasts, which are important cells involved in cell proliferation and migration. Considering the overall results, PZ1E and PZ2E scaffolds for future tissue regeneration and wound healing studies seem promising. As part of our ongoing research, the detailed cytokine and growth factor expression analysis along with the incorporation of ECM in 3D models will be carried out to strengthen the results to develop an optimized biomaterial.

## Supporting Information Available

Quantification of Zn in the PZ and PZE fibers (PDF)
