# Supplementary material for: Enhanced Cell Proliferation, Migration, and Fibroblast Differentiation with Electrospun PCL–Zinc Scaffolds Coated with Fibroblast-Derived ECM
Source: ACS Omega. 2025 Jan 28;10(5):4427–41. doi: 10.1021/acsomega.4c07504 (PMC11822518; doi:10.1021/acsomega.4c07504)
Supplement: Supplementary file 1 — ao4c07504_si_001.pdf [file ao4c07504_si_001.pdf]

Supporting Information

Enhanced Cell Proliferation, Migration, and Fibroblast Differentiation with Electrospun PCL-Zinc Scaffolds Coated with Fibroblast Derived ECM

Alexis Moody[1], Narayan Bhattarai[2*]

[1] Department of Applied Science and Technology, North Carolina A&T State University, Greensboro, NC, 27411, USA

[2] Department of Chemical, Biological, and Bioengineering, North Carolina A&T State University, Greensboro, NC, 27411, USA

* Correspondence to: Narayan Bhattarai, Department of Chemical, Biological and Bioengineering, North Carolina A&T State University, Greensboro, NC, 27411, USA

Email: nbhattar@ncat.edu

Table S1: Zinc Concentration of PZ and PZE Scaffolds

The zinc (Zn) concentration in sterilized electrospun fibers was quantified using inductively coupled plasma optical emission spectroscopy (ICP-OES Optima 8300, Perkin Elmer, Shelton, Connecticut, USA). Samples were prepared by digesting the fibers with concentrated nitric acid (67-70%, Fisher Scientific) and hydrofluoric acid (48-51%, VWR Chemicals) using an automated sequential microwave digester , MARS 6 (CEM Microwave Technology Ltd., North Carolina, USA). As, expected, all PZ fibers except the control PZ0 contained Zn. PZE fibers, which underwent surface modification, retained approximately 50% less Zn compared to the unmodified PZ fibers as seen in Table S1.

Fiber Name	Zn Concentration (wt%)
Control (PZ0)	0
PZ1	0.02 ± 0.007
PZ1E	0.01 ± 0
PZ2	0.14 ± 0.021
PZ2E	0.07 ± 0.021
